# A Case Study Demonstrating Applications of ChatGPT in the Clinical Management of Treatment-Resistant Schizophrenia

**DOI:** 10.7759/cureus.38166

**Published:** 2023-04-26

**Authors:** Pearl Valentine Galido, Saloni Butala, Meg Chakerian, Davin Agustines

**Affiliations:** 1 Medicine, College of Osteopathic Medicine of the Pacific, Western University of Health Sciences, Pomona, USA; 2 Psychiatry, Olive View-University of California Los Angeles Medical Center, Sylmar, USA

**Keywords:** standard of care, psychosis, antipsychotics, artificial intelligence in medicine, chatgpt, treatment-resistant schizophrenia

## Abstract

Chat Generative Pre-trained Transformer, also known as ChatGPT, is a new artificial intelligence (AI) program that responds to user inquiry with discourse resembling human language. The range of ChatGPT capabilities caught the interest of the medical world after it demonstrated its ability to pass medical boards examinations. In this case report, we present the clinical treatment of a 22-year-old male diagnosed with treatment-resistant schizophrenia (TRS) and compare the medical management suggested by ChatGPT to current standards of care in order to assess the program’s ability to identify the disorder, evaluate potential medical and psychiatric work-up, and develop a treatment plan addressing the distinct nuances of our patient. In our inquiry with ChatGPT, we found that it can accurately identify our patient as having TRS and order appropriate tests to methodically rule out alternative causes of acute psychosis. Furthermore, the AI program suggests pharmacologic treatment options including clozapine with adjuvant medications, and nonpharmacologic treatment options including electroconvulsive therapy (ECT), repetitive transcranial magnetic stimulation (rTMS), and psychotherapy which align with current standards of care. Lastly, ChatGPT provides a comprehensive list of side effects associated with antipsychotics and mood stabilizers used to treat TRS. We found both potential for and limitations in the clinical application of ChatGPT to assist in the assessment and management of complex medical conditions. Overall, ChatGPT may serve as a powerful tool to organize medical data in a meaningful and palatable format for medical professionals to reference during patient care.

## Introduction

Chat Generative Pre-trained Transformer (ChatGPT) is an artificial intelligence (AI) program developed by OpenAI that was launched on November 30, 2022, and has since become a cultural phenomenon [1]. ChatGPT utilizes a large database of information to respond to user inquiries with language replicating human discourse. Thus far, the program has demonstrated its capacity to perform in medicine by successfully passing the United States Medical Licensing Examination (USMLE) Step 1, Step 2 clinical knowledge (CK), and Step 3 medical board examinations [1,2]. Additionally, the program has proven to create differential diagnosis lists with 93.3% accuracy of the actual disease when prompted with common clinical vignettes [3]. These processes demonstrate the ability of ChatGPT to match textbook knowledge to well-established diseases. Some propose that ChatGPT may serve the medical community by collecting information from electronic medical records, organizing content for literature reviews, connecting diagnoses with billing codes, and other data management services [4,5]. By taking advantage of AI’s ability to recognize and organize data, medical practitioners may focus more energy on patient care [6]. 

ChatGPT may have a role in optimizing treatment for complex diseases in psychiatry like treatment-resistant schizophrenia (TRS). Approximately 20%-30% of patients diagnosed with schizophrenia do not respond to antipsychotic medications [7]. If they fail to respond to at least two antipsychotic trials, these patients are described as having TRS [8]. These patients may require more potent antipsychotics, like clozapine, the only FDA-approved medication for TRS [8,9]. However, it may be associated with a greater risk of agranulocytosis, sialorrhea, myocarditis, cardiomyopathy, weight gain, sedation, and constipation [10]. Even then, clozapine has only demonstrated beneficial impacts on approximately one-third of this treatment-resistant population [7]. The pharmacologic management of TRS is challenging and poses an opportunity for ChatGPT to use extensive foundational knowledge of medicine alongside emerging data to provide pertinent feedback to clinician inquiry. Additional adjuvants to medication include electroconvulsive therapy (ECT), repetitive transcranial magnetic stimulation (rTMS), deep brain stimulation, expressive therapy, talk therapy, exercise therapy, cognitive behavioral therapy, and hallucination-focused integrative therapy [8,11-13]. When considering alternate or adjuvant treatment modalities, ChatGPT might provide evidence-based treatment recommendations tailored to patients’ needs, history, and preferences. TRS carries a significant clinical and economic burden on patients, families, and society [14,15]. Many studies suggest that patients with TRS have more severe symptoms, earlier age of diagnosis, and worse quality of life compared to their treatment-responsive counterparts [8]. TRS has been shown to have higher rates of hospitalization, health resource utilization, substance abuse, unemployment, aggression, and suicidality [14,15]. The AI platform ChatGPT may curate data to assist healthcare practitioners in providing efficient, personalized, and evidence-based care for complex medical conditions like TRS. 

In this case report, we present ChatGPT with the clinical vignette of a 22-year-old male diagnosed with TRS. Our aim is to compare the standard of care of TRS used by clinicians to that of ChatGPT in order to assess its ability to recognize TRS and provide accurate treatment plans. Furthermore, we aim to assess the function of ChatGPT in assisting physicians in the clinical assessment and management of complex medical cases.

## Case presentation

We report the case of a 22-year-old male with a six-year history of schizophrenia who was admitted to the inpatient psychiatric service with acute psychosis. He was admitted for unprovoked aggression secondary to paranoid ideation, delusions, and auditory hallucinations. This patient had multiple inpatient encounters due to refractory schizophrenia that was non-responsive to a variety of treatment regimens. Multiple medication failures resulted in a previously established combination treatment regimen involving clozapine, olanzapine, lithium, and valproate. Prior to this hospitalization, the patient was reported to have been non-adherent with his medication for at least four days, resulting in the decompensation of his TRS. 

Over the course of his care, the patient was identified to have TRS which required a well-balanced combination of antipsychotic medications and mood stabilizers. In the psychiatric emergency department, the clinical team utilized a series of labs - complete blood count (CBC) with differential, complete metabolic panel (CMP), thyroid stimulating hormone (TSH) level, vitamin B12 level, HIV reactivity, syphilis reactivity, and urine drug screen - in order to rule out alternative causes of acute psychosis. The lab results for this patient were within normal ranges. The patient was restarted on clozapine 25 mg two times per day, olanzapine 15 mg two times per day, valproate 1000 mg three times per day, and lithium 600 mg two times per day. Due to medication nonadherence, clozapine was slowly titrated up to the previously established therapeutic dose of 300 mg two times per day. The patient required a weekly CBC with differential to monitor for agranulocytosis. Additionally, lithium and valproate blood levels were monitored to ensure their serum concentrations were within the narrow therapeutic range. 

After one week, we cross-titrated antipsychotics by slowly tapering the patient off of olanzapine and increasing the dosage of clozapine. As the olanzapine dose decreased, the patient began demonstrating increased paranoia and unprovoked aggression toward staff and peers. Additionally, the patient began displaying signs of clozapine-induced sialorrhea. To address both the agitation and sialorrhea, we increased the olanzapine dosage to utilize its antihistamine and anticholinergic properties. Over the course of three weeks, we identified an optimal therapeutic regimen including clozapine 300 mg two times per day, olanzapine 10 mg two times per day, valproate 1000 mg three times per day, and lithium 600 mg two times per day. This regimen resulted in an overall decrease in the frequency of the patient’s auditory hallucinations, delusions, and paranoia.

In order to evaluate the utility of ChatGPT in the medical and psychiatric care of a patient with TRS, we presented ChatGPT with parameters based on this clinical presentation. We asked ChatGPT 23 open-ended questions over a series of four individual chat discussions (Table 1). Through these encounters, we analyzed the capacity of ChatGPT to identify medical and psychiatric conditions, propose differential diagnoses, suggest comprehensive assessment plans, and provide integrated treatment plans. We had an additional fifth encounter with two questions to assess ChatGPT's ability to organize treatment options and provide references regarding standards of care for TRS.

**Table 1:** List of ChatGPT encounters regarding clinical case including parameters and topics of interest In four separate chat discussions with ChatGPT, we varied parameters and analyzed ChatGPT’s curated content for different topics of interest regarding the case of a 22-year-old male with TRS. Symptoms were delusional thinking, responding to internal stimuli, and unprovoked aggression. ChatGPT: Chat Generative Pre-trained Transformer; TRS: Treatment-resistant schizophrenia

Encounter	Parameters	Topic of Interest
One	Age, Gender, Symptoms	Identification of disease and assessment
Two	Age, Gender, Symptoms	Treatment
Three	Age, Gender, Symptoms, Past Medical History, Medications	Identification of disease, assessment, and treatment
Four	Age, Gender, Symptoms, Medications	Inpatient clinical medication management and medication adverse effects

## Discussion

We have described the case of a 22-year-old male with TRS who required careful consideration and balance of atypical antipsychotics and mood stabilizers. This clinical case allows us to challenge ChatGPT with the complexity involved in managing TRS through a series of encounters. These encounters assessed the functional value of ChatGPT assisting healthcare providers in identifying an appropriate therapeutic regimen, optimizing medication dosages, and managing adverse effects.

Encounter one

**Figure 1:**
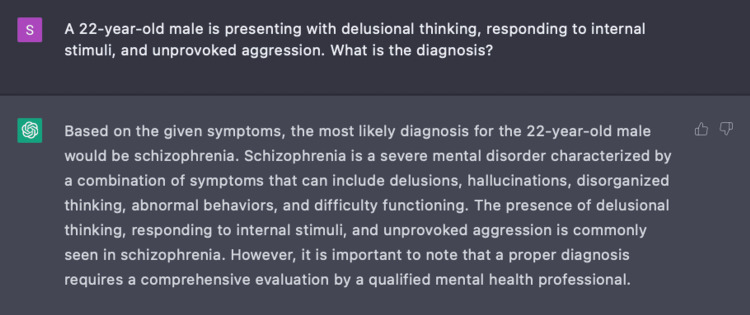
Using ChatGPT to determine a medical condition ChatGPT was able to identify the medical condition based on the initial three parameters (age, gender, and symptoms) with which it was presented.

In Encounter One, we prompted ChatGPT with a series of nine questions based on three parameters: age, gender, and three pertinent symptoms of delusional thinking, responding to internal stimuli, and unprovoked aggression. With only these parameters given, we engaged ChatGPT to determine a primary diagnosis for this patient (Figure 1). 

**Figure 2:**
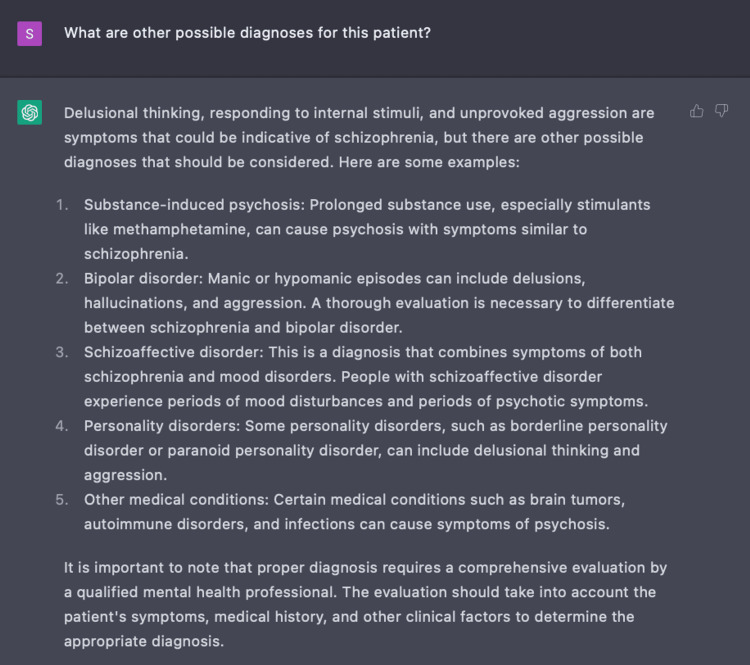
Using ChatGPT to develop differential diagnoses ChatGPT was able to generate a comprehensive list of differentials based on our patient’s presenting symptoms of delusional thinking, responding to internal stimuli, and unprovoked aggression.

**Figure 3:**
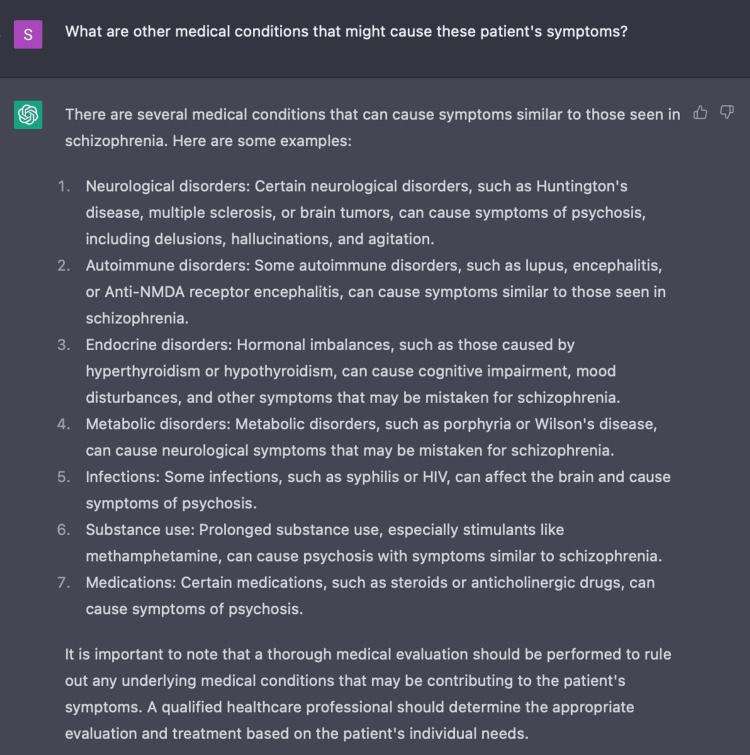
Using ChatGPT to develop non-psychiatric differential diagnoses ChatGPT was able to identify non-psychiatric causes of acute psychosis based on our patient’s presenting symptoms of delusional thinking, responding to internal stimuli, and unprovoked aggression.

In our inquiry with ChatGPT, we also found that the AI program is able to present differential diagnoses, including schizophrenia, for a patient presenting with positive symptoms of acute psychosis as exhibited in Figure 2. When further questioned, ChatGPT demonstrated clinical value by populating a list of possible non-psychiatric causes of acute psychosis including porphyria and encephalitis (Figure 3). This exhibits ChatGPT’s ability to create a broad differential which is imperative in the initial evaluation of a disease.

**Figure 4:**
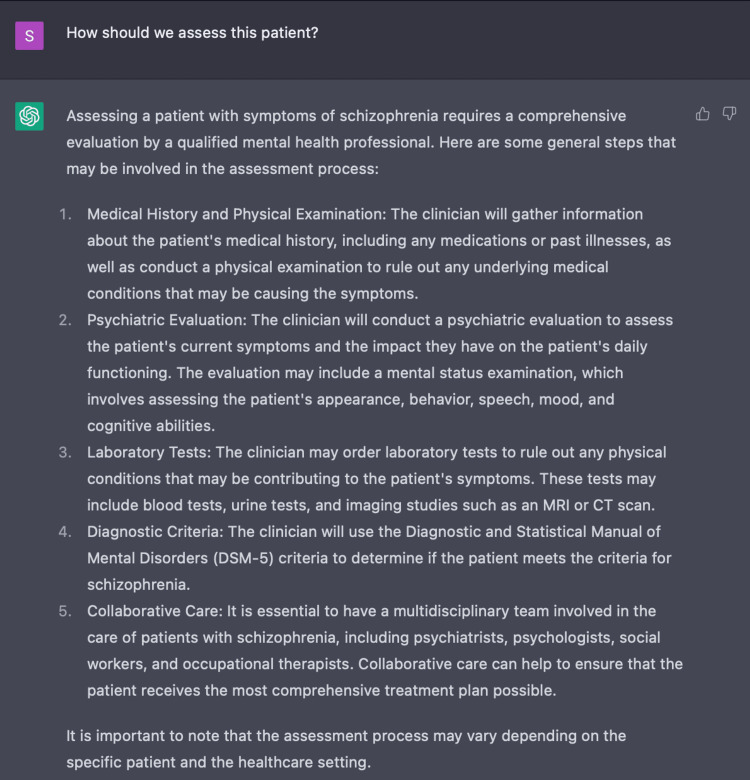
Using ChatGPT to develop an initial plan of assessment ChatGPT’s response to inquiry about initial assessment of a patient presenting with positive symptoms of acute psychosis.

In further discussion with ChatGPT to determine an appropriate plan of assessment, ChatGPT initially presented a relatively non-specific plan with suggestions including: gathering a medical history, performing a physical and mental status examination, considering additional labs and imaging, and consulting healthcare providers (Figure 4). In response to ChatGPT’s vague response, our questions evolved from “how should we assess this patient,” to “what should I, as a healthcare provider, do to assess this patient.” Despite our inquiry becoming more specific, the response of ChatGPT remained as non-specific as the answers in Figure 4. 

In order to gain further insight on appropriate assessment for this patient case, we asked “What labs/imaging should be ordered for this patient and why?” Initially ChatGPT suggested: CBC, CMP, thyroid function tests, and imaging studies such as CT or MRI. However, this list is incomplete compared to the comprehensive clinical laboratory work-up for acute psychosis [16]. Therefore, we asked ChatGPT to expand upon additional appropriate labs by asking, “What other labs should be ordered at this time?” In response, ChatGPT included: Vitamin B12 levels, serology tests, toxicology, and urinalysis. On one hand, ChatGPT did eventually provide a comprehensive laboratory workup for acute psychosis, demonstrating its ability to choose tests that assess for the various medical and psychiatric causes of psychosis. This function is valuable because it prevents oversight of key details revealed by certain labs that may help to identify underlying causes of acute psychosis like illicit drug use, alcohol-induced encephalopathy, and infection [16]. On the other hand, it required additional user inquiry and provocation to complete this task, demonstrating that users need a broader clinical understanding of psychosis than available to ChatGPT. This finding may prove to be a limitation of ChatGPT in testing for medical conditions in that ChatGPT alone cannot create comprehensive standardized plans without additional probing, thus highlighting the importance of utilizing clinical judgment to drive patient management.

Encounter two

**Figure 5:**
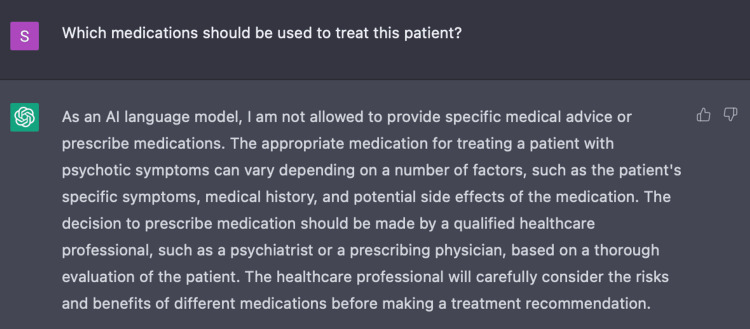
ChatGPT disclaimer ChatGPT acknowledges one of its own limitations on providing medical advice and prescribing medications. Rather, it advises supervision under a medical provider.

**Figure 6:**
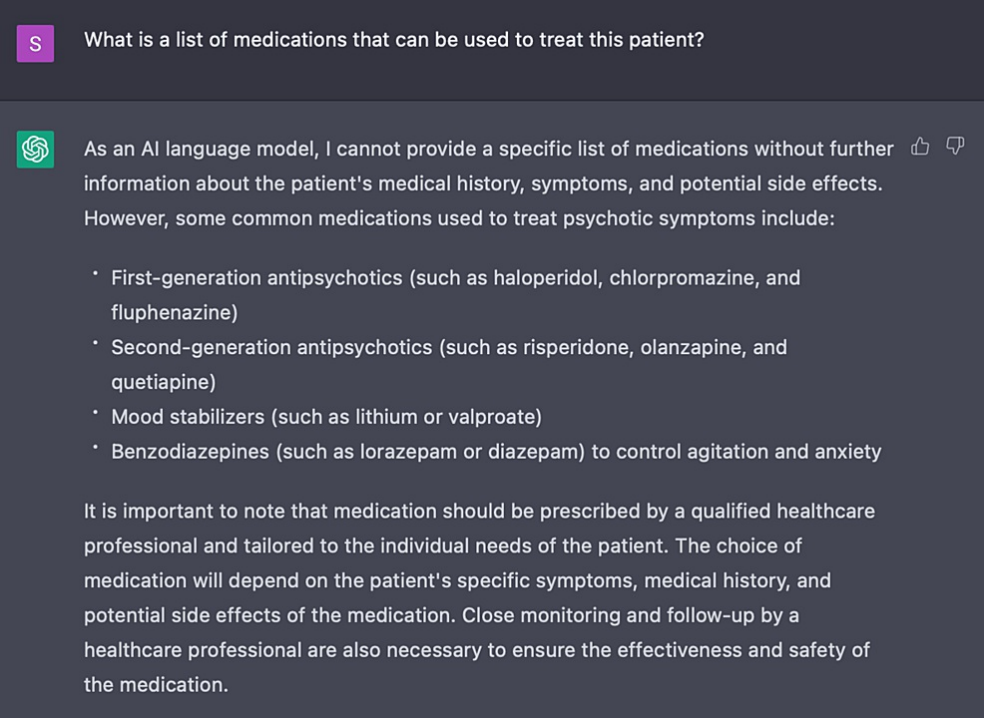
Using ChatGPT to determine appropriate medications for acute psychosis ChatGPT recommended medications based on the following parameters: age, gender and symptoms of delusional thinking, responding to internal stimuli, and unprovoked aggression.

In Encounter Two, we assessed the capacity of ChatGPT to determine an appropriate treatment plan for this patient case based on the initial three parameters with a series of three questions. Initially, ChatGPT provided a non-specific list of recommendations including: antipsychotic medications, psychotherapy, and hospitalization. In simply asking ChatGPT to recommend medications for this patient, ChatGPT establishes its own limitations stating that as an AI language model it is unable to provide specific medical advice or prescribe medications (Figure 5). When specifically asking ChatGPT for a list of medications, it provides commonly used medications such as first-generation antipsychotics, second-generation antipsychotics, mood stabilizers, and benzodiazepines (Figure 6). However, ChatGPT also mentions the necessity of additional parameters such as the patient's medical history, symptoms, and side effects to generate a more tailored list. While making such suggestions, ChatGPT recommends appropriate input from healthcare providers to discern risks and benefits for each patient. 

Encounter three

**Figure 7:**
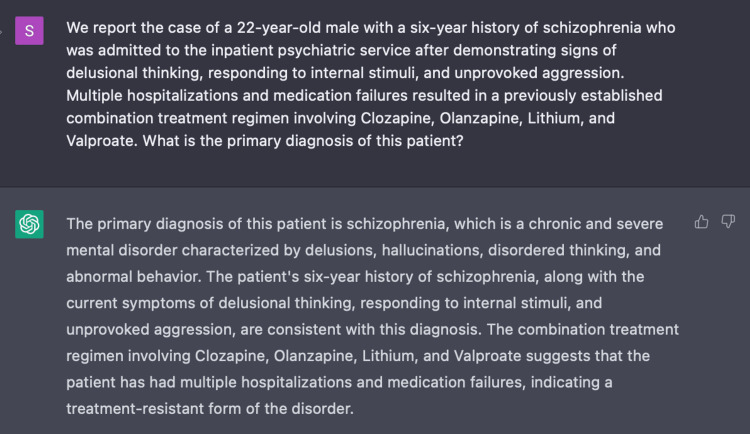
ChatGPT’s recognition of treatment-resistant schizophrenia ChatGPT recognizes TRS, a more complex form of schizophrenia, based on a clinical vignette with the following five parameters: age, gender, past medical history, medications, and pertinent symptoms of delusional thinking, responding to internal stimuli, and unprovoked aggression.

In Encounter Three, we prompted ChatGPT with a more formal clinical vignette including a series of three questions with the following five parameters: age, gender, past medical history, medications, and three pertinent symptoms. Once again, we assessed the capacity of ChatGPT to provide a diagnosis and assessment plan. With greater user input regarding patient history, ChatGPT was able to distinguish our presented case as a treatment-resistant form of schizophrenia (Figure 7). Not only that, it expanded its assessment to incorporate: family history, neuropsychological testing, and social history.

**Figure 8:**
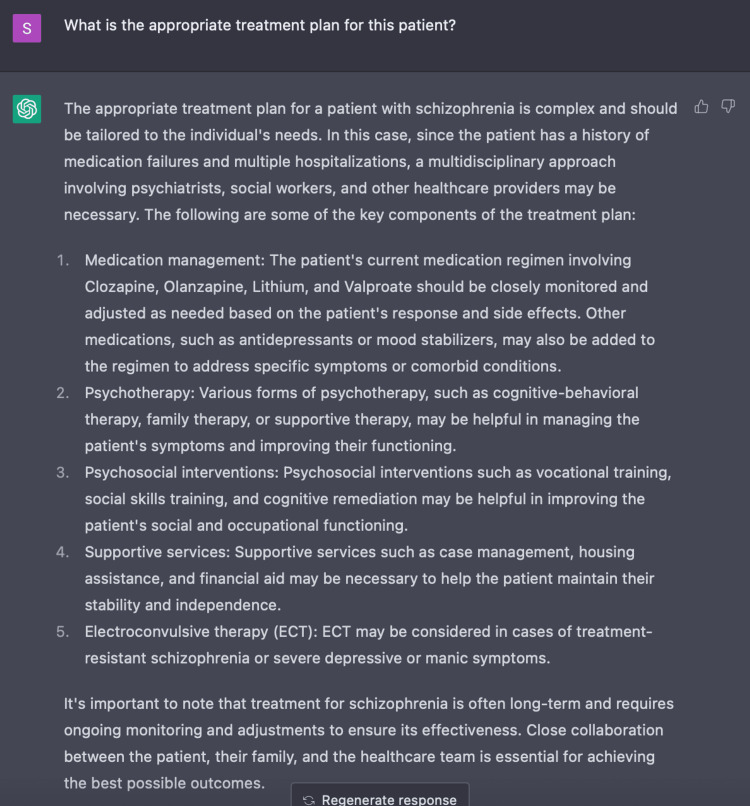
Using ChatGPT to create a treatment plan for treatment-resistant schizophrenia ChatGPT was able to suggest a holistic plan in the management of TRS including pharmacologic and non-pharmacologic approaches.

Additionally, we evaluated the clinical use of ChatGPT in developing a plausible treatment plan for this patient. ChatGPT responded with a full spectrum of possible modes of care by discussing medication management, psychotherapy, psychosocial interventions, supportive services, and ECT as exhibited in Figure 8. When given additional parameters, ChatGPT provides a more comprehensive and curated treatment plan in comparison to when only given the initial three parameters as in Encounter Two. With ChatGPT’s ability to discern this case of TRS, it included the vital recommendation of ECT. ECT is an evidence-based, nonpharmacologic treatment approach in patients with considerable refractory schizophrenia [8,17]. Furthermore, given the significant clinical and economic burden placed by TRS on patients, families, and society, ChatGPT highlights key social factors in the management of TRS [14,15]. ChatGPT made suggestions involving vocational and social skills training to improve social and occupational functioning as well as case management, housing assistance, and financial aid to provide crucial support for these patients (Figure 8). Due to the complexity of TRS, it is imperative that we optimize treatment plans by incorporating psychosocial training and supportive services alongside mainstay treatment regimens.

Encounter four

**Figure 9:**
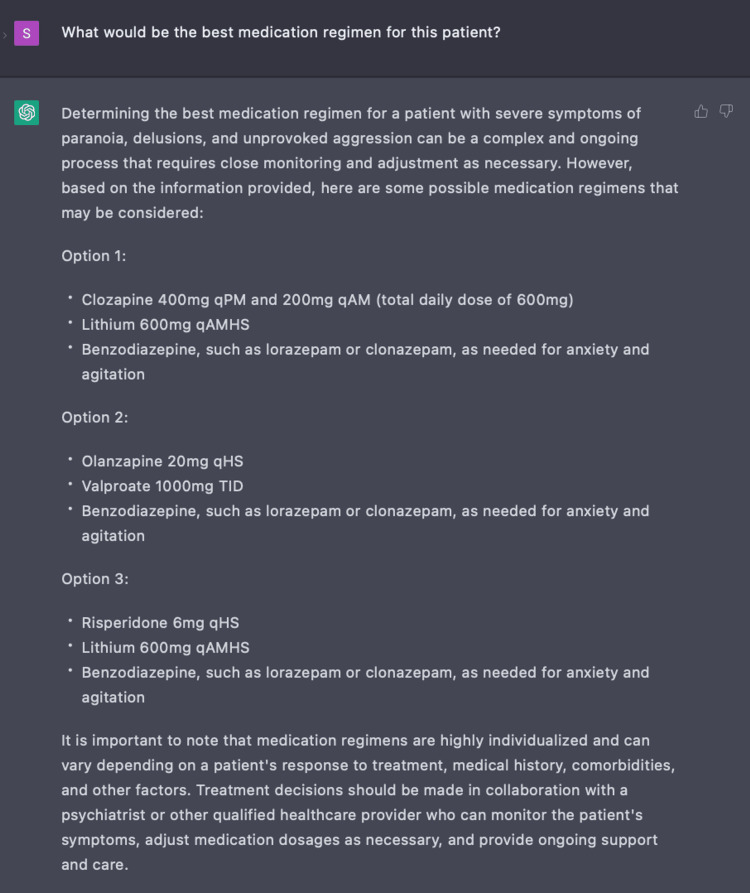
Use of ChatGPT in developing medication regimen for treatment-resistant schizophrenia ChatGPT was able to suggest various combinations and dosing of multidrug therapy for the management of treatment-resistant schizophrenia.

In Encounter Four, we gathered insight on the value of ChatGPT in inpatient management for this patient case by asking a series of five questions with the following four parameters: age, gender, symptoms, and medications. During the course of our patient’s care, he continued to display signs of unprovoked aggression and paranoia while on the psychiatric inpatient ward. Thus, we wished to compare our clinical management of this patient based on standardized guidelines to treatment recommendations made by ChatGPT [8,17]. Through a series of probing questions, ChatGPT responded with clozapine, ECT, rTMS, and psychosocial therapy which correlates with the current guidelines [8,12,17]. It even goes on to suggest non-pharmacologic options like supplements, acupuncture, and yoga. When further prompted to provide examples of pharmacologic treatment, ChatGPT provided combinations and doses of clozapine with a benzodiazepine or valproate, and olanzapine with lithium which are all combinations listed in the management of TRS as exhibited in Figure 9 [5,8,17]. For this inquiry, ChatGPT provided efficient evidence-based multidrug therapeutic regimens for TRS. However, ChatGPT acknowledged its own limitations by providing a disclosure to defer to a psychiatrist and healthcare team for definitive therapy.

**Figure 10:**
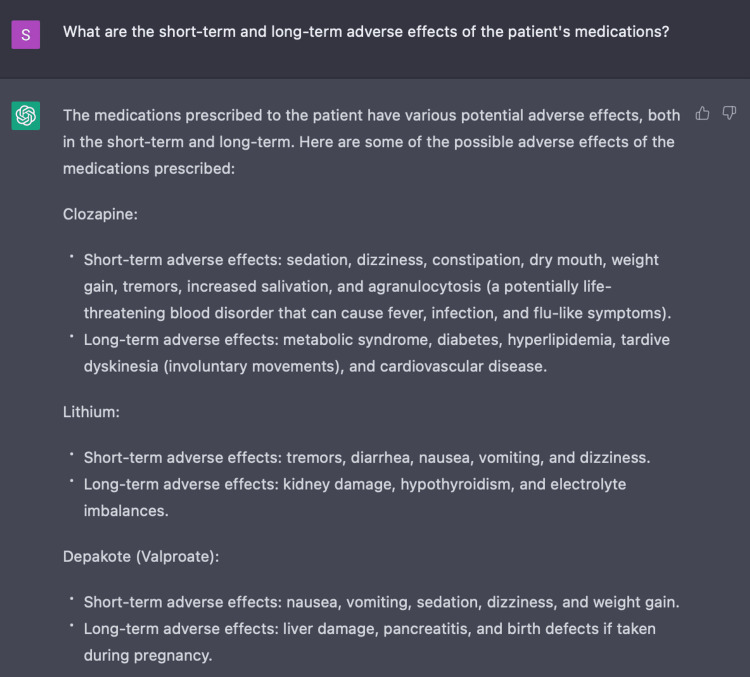
Use of ChatGPT in identifying medication adverse reactions ChatGPT generates a list of short-term and long-term adverse reactions associated with medications listed within the indicated therapeutic treatment regimen.

**Figure 11:**
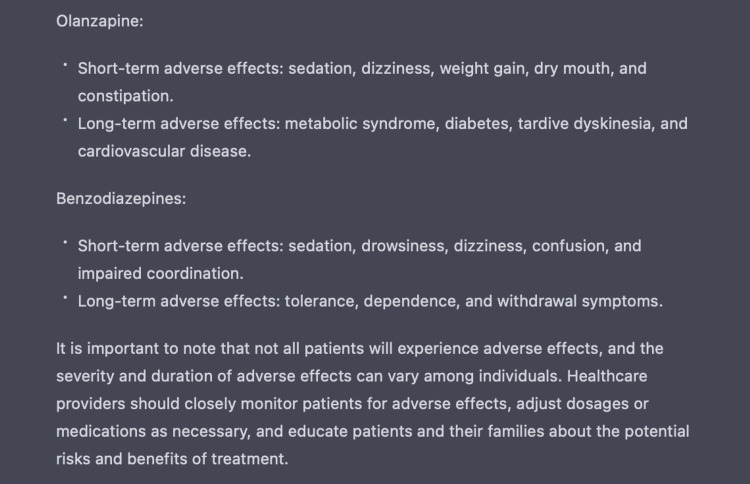
Use of ChatGPT in identifying medication adverse reactions ChatGPT generates a list of short-term and long-term adverse reactions associated with medications listed within the indicated therapeutic treatment regimen.

The use of multiple antipsychotics supplemented with antidepressants and antiepileptics can result in many side effects [10]. Thus we also evaluated the function of ChatGPT to generate a list of side effects to monitor for said medications - some of which include severe adverse reactions like agranulocytosis and organ toxicity - as demonstrated in Figures 10-11 [10]. This feature proves to be important in that it can provide healthcare providers with clinical insight on monitoring for adverse reactions and therefore act as a secondary checkpoint.

Clinical use of ChatGPT in assessment and management of treatment-resistant schizophrenia

Overall, ChatGPT has proven to be a useful tool in synthesizing data and reporting information in a digestible form. In the future, this program may be useful in providing clinicians with concise background information, workup, and management of complex medical conditions.

As exhibited, ChatGPT proves that when provided with specific details, it is able to recognize TRS and suggest laboratory studies for diagnosing. Not only that, but it can also provide concise treatment recommendations, offer pharmacologic and nonpharmacologic treatments, and identify side effects of medications based on current standards of care and published literature [2,3,8,17]. Specifically, it can suggest treatment plans that address the very complex disease of TRS. One of the major difficulties to treating TRS is that there are many adjuvant medications to clozapine and there is mixed evidence for the efficacy of each combination [8,17]. 

**Figure 12:**
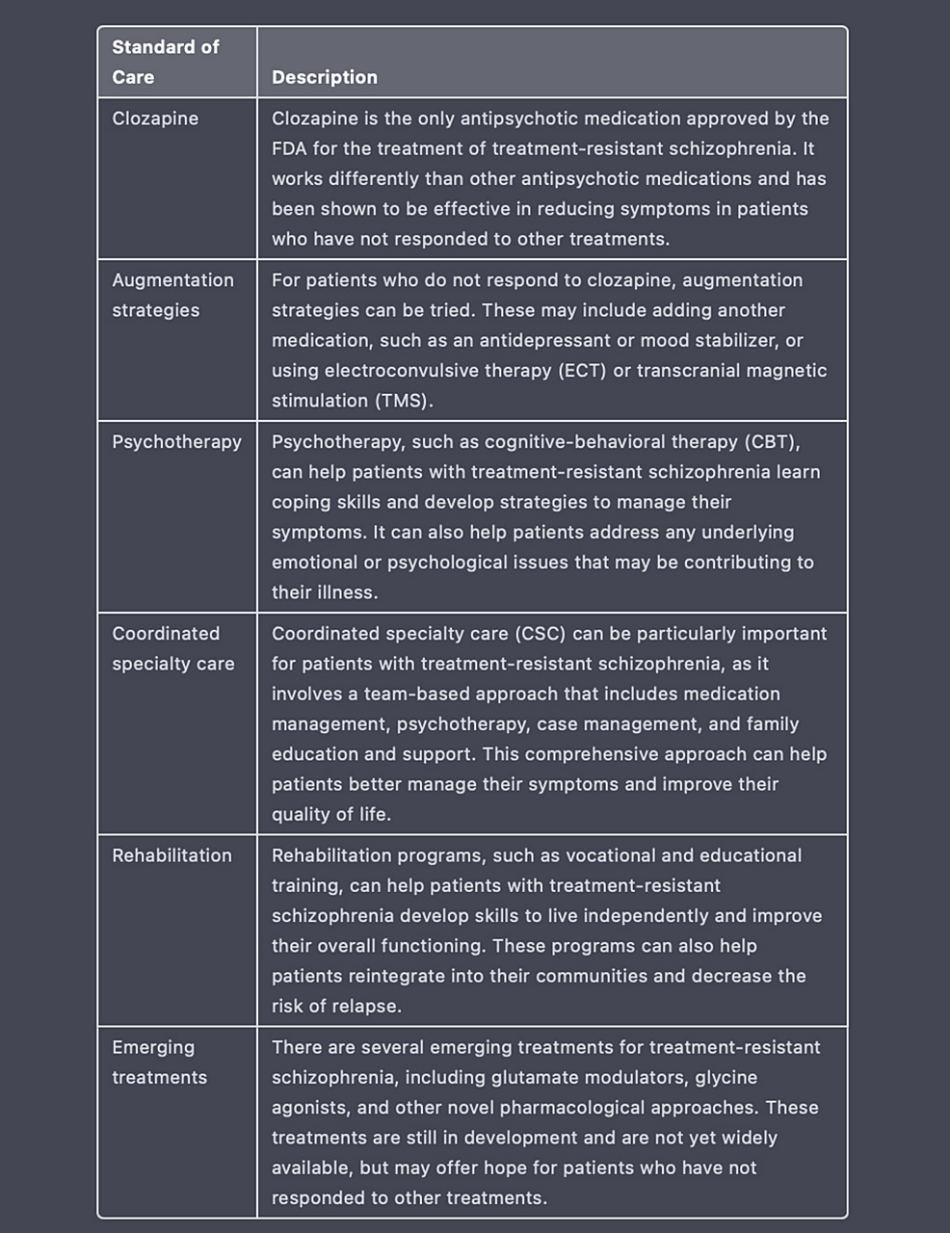
Using ChatGPT to create a table ChatGPT generates a table detailing the current standards of care and newly emerging treatments for TRS.

**Figure 13:**
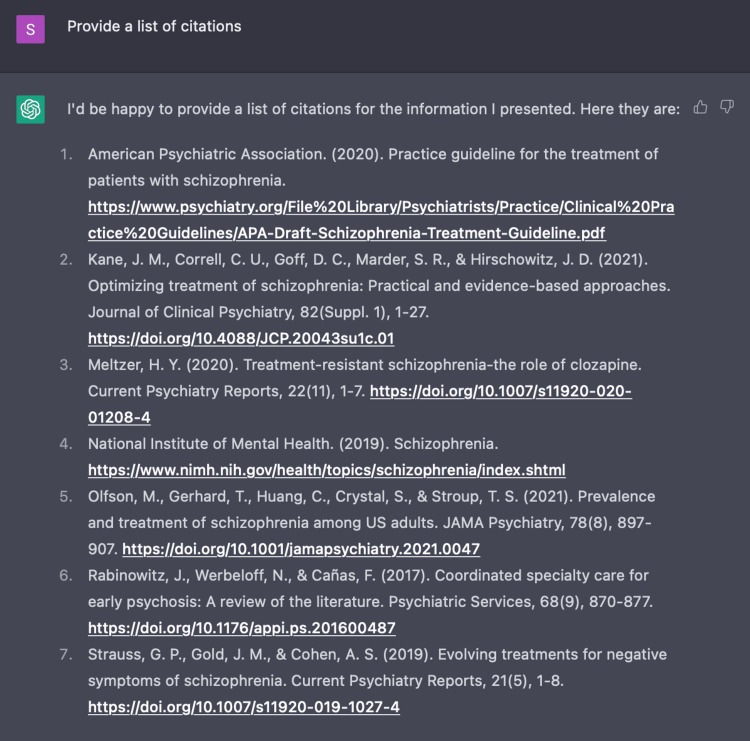
ChatGPT provides reference list After ChatGPT generated a table with current standards of care for treatment-resistant schizophrenia, we prompted the AI program to provide a reference list.

ChatGPT organizes information and provides data in a palatable format for medical practitioners to access quickly and reliably when compared to the practice of searching through various databases like Pubmed, UpToDate, and Medscape with scattered information. For example, when we utilized an additional fifth encounter to prompt ChatGPT to create a table on current standards of care for TRS, it presents us with the detailed table exhibited in Figure 12. After creating this table, ChatGPT is also able to create a reference list based on the given information as displayed in Figure 13. Considering the accuracy of ChatGPT’s recommendations regarding TRS, this AI program may prove to be useful in identifying and managing other complex diseases. Additionally, ChatGPT may ultimately serve to decrease the clinical and economic burden of patients who may be left untreated or not optimally treated by providing healthcare providers with diverse modes of treatment based on a clinical vignette.

Limitations of ChatGPT in medicine

One of the major limitations to ChatGPT is that it relies on variables determined by user input in order to produce a response. If users input data incorrectly or suggest parameters that limit data collection, ChatGPT is forced to produce inaccurate information. In the case of medicine where weight, medication dosage or medical abbreviation are all factors in patient care, a simple human error such as an incorrect decimal point or misspelling may result in an unintentional inaccurate output from ChatGPT. It requires human knowledge and understanding to recognize these errors and proceed with caution when utilizing AI suggestions. 

ChatGPT also lacks the ability to ask users to clarify an input instruction. It may list parameters it needs to expand an answer, but it will not facilitate discussion or explicitly request the alteration of an existing inquiry necessary to formulate an accurate response. Without this intrinsic property, ChatGPT loses an error check option that users could modify for a more comprehensive response. This may prove to be a barrier to novice users and novice medical professionals. 

Another limitation of ChatGPT is the lack of clinical context. It has been demonstrated that ChatGPT can collect medical information and suggest treatments based on published clinical data. It may not, however, prove to be as adaptable in unpublished clinical contexts. Much of clinical practice is based on the personal experience of the individual provider which is not readily available in published literature for ChatGPT. In these instances, patients rely on the clinical expertise of medical professionals to decipher the minutiae involved in managing their care.

Lastly, unlike common medical databases like PubMed or UpToDate which use in-text citations and explicitly list references, ChatGPT must be prompted to supply a reference list. Therefore, when considering the information presented by ChatGPT, it is imperative that medical professionals request a reference list to assess whether or not the information is derived from trusted and reliable sources. Additionally, it is important to recognize that different regions of the world may have different guidelines for management of certain medical conditions depending on epidemiology, socioeconomics, and psychosocial factors. At this time, we are unable to assess whether or not ChatGPT’s recommendations can adhere to region-specific guidelines for care.

## Conclusions

In this case report, we found evidence that ChatGPT is able to perform intricate tasks relevant to handling complex medical information. To assess ChatGPT’s performance against clinical questions, we tested its assessment and treatment plan for a 22-year-old male diagnosed with TRS and compared the result with the current standards of evaluation and treatment. Given the parameters of age, gender, past medical history, medications, and three pertinent symptoms of delusional thinking, responding to internal stimuli, and unprovoked aggression, ChatGPT was able to recognize our patient as having TRS. Additionally, ChatGPT was able to suggest comprehensive laboratory workup to determine medical and psychiatric causes of psychosis. Finally, ChatGPT was able to suggest a holistic treatment plan including pharmacologic and non-pharmacologic forms of treatment for TRS which align with current clinical standards of care. 

ChatGPT was able to provide adequate information for clinicians to approach TRS, however, there are also some pertinent limitations on the clinical use of ChatGPT. For one, the quality of AI-generated responses heavily relies on the quality of information input into the system. Additionally, it lacks the ability to request edits to input errors and may be programmed to produce inaccurate information. ChatGPT also lacks in-text citation which decreases transparency of AI-generated responses, thus requiring users to request a reference list. While ChatGPT produced appropriate assessment and treatment plans for TRS based on clinical standards of care, it was unable to fully address the complexities of a real-world case in regard to medication failure and side effect profile without clinician evaluation. Thus, clinical care supported by ChatGPT requires the expertise of a trained and knowledgeable medical professional. Despite its limitations, ChatGPT has demonstrated promising functions in recognizing complex diseases, creating differential diagnoses, proposing appropriate laboratory workups, and suggesting comprehensive treatment plans. In the future, we should also assess the function of ChatGPT in healthcare quality improvement initiatives such as decreasing the administrative burden on physicians by collecting electronic medical record data and implementing triage for varying degrees of illness. These functions may result in increasing efficiency and safety in patient care. As ChatGPT continues to expand and update its knowledge base, we are optimistic that it may one day play a large role in assisting healthcare practitioners in providing higher quality of care. 
